# Prevalence, awareness and control of diabetes in the Seychelles and relationship with excess body weight

**DOI:** 10.1186/1471-2458-7-163

**Published:** 2007-07-19

**Authors:** David Faeh, Julita William, Luc Tappy, Eric Ravussin, Pascal Bovet

**Affiliations:** 1 University Institute for Social and Preventive Medicine (IUMSP), Lausanne, Switzerland; 2 Department of Physiology, University of Lausanne, Lausanne, Switzerland; 3 Ministry of Health, Victoria, Republic of Seychelles; 4 Pennington Biomedical Research Centre, Baton Rouge, LA, USA

## Abstract

**Background:**

The evidence for a "diabesity" epidemic is accumulating worldwide but population-based data are still scarce in the African region. We assessed the prevalence, awareness and control of diabetes (DM) in the Seychelles, a rapidly developing country in the African region. We also examined the relationship between body mass index, fasting serum insulin and DM.

**Methods:**

Examination survey in a sample representative of the entire population aged 25–64 of the Seychelles, attended by 1255 persons (participation rate of 80.2%). An oral glucose tolerance test (OGTT) was performed in individuals with fasting blood glucose between 5.6 and 6.9 mmol/l. Diabetes mellitus (DM), impaired fasting glucose (IFG) and impaired glucose tolerance (IGT) were defined along criteria of the ADA. Prevalence estimates were standardized for age.

**Results:**

The prevalence of DM was 11.5% and 54% of persons with DM were aware of having DM. Less than a quarter of all diabetic persons under treatment were well controlled for glycemia (HbA1c), blood pressure or LDL-cholesterol. The prevalence of IGT and IFG were respectively 10.4% and 24.2%. The prevalence of excess weight (BMI ≥ 25 kg/m^2^) and obesity (BMI ≥ 30 kg/m^2^) was respectively 60.1% and 25.0%. Half of all DM cases in the population could be attributed to excess weight.

**Conclusion:**

We found a high prevalence of DM and pre-diabetes in a rapidly developing country in the African region. The strong association between overweight and DM emphasizes the importance of weight control measures to reduce the incidence of DM in the population. High rates of diabetic persons not aware of having DM in the population and insufficient cardiometabolic control among persons treated for DM stress the need for intensifying health care for diabetes.

## Background

It is estimated that diabetes mellitus (DM) accounts currently for 5.2% of all deaths worldwide [1]. The number of people with DM is expected to double from 175 million in 2000 to 353 million in 2030 [2]. The largest increase is expected to occur in developing countries, with 305 million individuals likely to have DM by 2030 [2].

The prevalence of DM in adults varies markedly between different populations, e.g. 2.6% in Nigeria [3], 18% in Mauritius [4], and more than 50% in Pima Indians in the U.S. [5]. These differences have been related to unfavorable trends in factors such as overweight and sedentary habits, as demonstrated in longitudinal, ecological and migration studies [4-6] and to interactions between environmental and genetic factors when individuals become exposed to an obesogenic environment [4,7].

In developing countries, the prevalence of diabetes is markedly higher in urban than rural areas (e.g. 2.0% vs. 0.8% in Cameroon in 1999 or 14% vs. 5% in Egypt in 1995) [8,9]. There is also a gradient across socio-economic development stages, e.g. a prevalence of DM in African individuals of 2% in Cameroon, 9% in Jamaica, 11% in Trinidad and Tobago and 15% in Manchester [8,10], which further emphasizes the role of environment factors in populations of same genetic origin. However, data on the prevalence, awareness and control of DM remain limited in the African region.

Based on a population-based survey, we assessed the prevalence, awareness and control of diabetes (DM) in the Republic of Seychelles, a rapidly developing country in the African region. We also examined the relationship between body mass index, fasting serum insulin and DM and the proportion of all diabetic persons in the population that could be attributed to excess body weight.

## Methods

The Republic of Seychelles consists of over 100 islands located in the Indian Ocean about 2000 km east of Kenya and 2000 km north of Mauritius, in the African region. Approximately 90% of the population of Seychelles lives on the main island (Mahé) and most of the remaining population resides on two nearby islands (Praslin and La Digue). Although intermarriage has blurred racial differences in many Seychellois, it can be considered that approximately two thirds of the population is of predominantly African descent, 15% is of predominantly Caucasian, Indian or Chinese descent, and a fifth is mixed between these various groups. The population of Seychelles can be considered as fairly urbanized in view of the high density of the population and the facts that a large proportion of the population commutes to the capital for work and three quarters of workers are employed in services [11,12]. The national gross domestic product per capita, in real terms, rose from US$ 2927 in 1980 to US$ 5239 in 2004 [13], reflecting booming tourism and industrial fishing industries. In Seychelles, cardiovascular disease and AIDS currently account for 38% and 1% of all deaths, respectively [11]. Health care (inclusive medications) is provided free of charge to all inhabitants. The disease burden related to diabetes is significant in Seychelles (e.g. a majority of lower limb amputations and a third of all persons under hemodialysis are related to diabetes), although the precise diabetes-related disease burden has not been systematically determined yet.

A population-based examination survey for cardiovascular risk factors was conducted in 2004 under the auspices of the Ministry of Health of the Republic of Seychelles. The sampling frame consisted of a sex- and age- stratified random sample of the population aged 25–64. Eligible individuals were selected from a computerized database derived from population censuses (last in 2002) thereafter regularly updated by civil status authorities. Eligible participants were invited to participate through a personal letter requesting them to attend designated survey centers on a particular date, fasting, between 7:30 and 11:00 am and informing them that snacks would be provided on the study center. Participants were free to participate and gave informed written consent. The survey was approved by the research and ethical board of the Ministry of Health of the Republic of Seychelles.

A structured questionnaire was administered by trained survey officers. The questions assessed, among other items, if participants were "ever told by a doctor that they had DM" and if they "were currently under treatment for DM". If treatment was reported, participants were considered to have "known" DM. Family history of DM was defined for participants who reported DM among a first degree parents or siblings. Weight was measured with electronic scales to 0.2 kg precision (Seca, Hamburg, Germany) and height was measured with fixed stadiometers to 0.5 cm precision (Seca). BMI was calculated as weight divided by height squared (kg/m^2^). Blood pressure (BP) refers to the average of the second and third of three measurements (mercury sphygmomanometer, cuff size adjusted to arm circumference).

Venous blood glucose was measured with a Cholestec LDX, a point-of-care analyzer which is a reliable alternative to conventional laboratory devices [14]. The Cholestec instrument separates blood cells from plasma and measurements are therefore made on plasma. If glucose was ≥ 5.6 mmol/l, another measurement was carried out a few minutes later on capillary blood (Bayer, Ascentia Elite [15]) and the mean of both readings was used. Of note, the Ascentia Elite automatically adjusts reading to plasma values. The difference between the first measurement (Cholestec) and the second measurement (Ascentia Elite) was as small as -0.15 mmol/l. Individuals who had FBG ≥ 5.6 mmol/l and <7.0 mmol/l and were not aware of having DM were submitted to an oral glucose tolerance test (OGTT) using 75 g glucose dissolved in 300 ml water and capillary glucose (Ascentia Elite) was measured 120 minutes later (2hBG). Glycated hemoglobin (HbA1c) was measured in known or new cases of DM using a point-of-care analyzer (DCA 2000, Bayer). The DCA 2000 has been recommended for measurement of HbA1c outside of the laboratory [16].

Categories of impaired glucose regulation were based on the 2004 criteria of the American Diabetes Association [17]. DM was defined as plasma FBG ≥ 7.0 mmol/l, 2hBG ≥ 11.1 mmol/l or current history of antidiabetic medication. IFG refers to FBG of 5.6–6.9 mmol/l. IGT was defined as FBG <7.0 mmol/l and 2hBG of 7.8–11.0 mmol/l. Normal glucose tolerance (NGT) was defined as 2hBG <7.8 mmol/l. Normal fasting glucose (NFG) refers to FBG values <5.6 mmol/l. Since subjects with NFG were not tested for 2-hour glucose, we cannot determine DM based on 2hBG ≥ 11.1 mmol/l and NFG.

Serum was obtained within 2 hours of blood collection and immediately frozen to -20°C. Fasting serum insulin (FSI) was measured at the University of Lausanne using commercial RIA kits (LINCO Research Inc, Missouri, USA). HOMA-IR (homeostasis model assessment of insulin resistance) was calculated as [FSI (μU/ml) × FBG (mmol/l)]/22.5 [18]. Blood lipids were measured using standard methods (Hitachi 917 instrument and Roche reagents) and low-density lipoprotein cholesterol (LDL-C) calculated with the Friedewald formula.

Overall estimates in the population aged 25–64 were standardized to the new age distribution of the World Health Organization [19], using weighted "svy" commands in Stata. Differences were tested with the chi-square test and the t-test, respectively. For medians inter-quartile ranges were calculated. Increases in mean FSI across categories of impaired glucose regulation and BMI categories were tested with the Cuzik trend test. The association between DM and body mass index categories was analyzed with logistic regression adjusted for age and sex and weighted for the age-stratified sampling frame ("svylogit"). We used a model aggregating men and women since the interaction of BMI with sex was not significant. We calculated the proportions of all DM cases in the population that could be attributable to overweight and obesity (population attributable fraction, PAF) using the weighted "aflogit" command in Stata. PAF is conceptually computed as P(RR-1)/[1+P(RR-1)], where P is the prevalence of excess weight in the population [20] and RR is the risk ratio of the exposure (excess weight) on the outcome (DM). RR was estimated with the adjusted odds ratio (OR) derived from the multivariate logistic regression models and confidence intervals were based on asymptotic approximation [20]. Analyses were performed with Stata 8.2 and p values less than 0.05 were considered significant.

## Results

1255 out of 1565 (80.3%) eligible individuals participated. Age-standardized mean values and prevalence rates of selected characteristics are presented in Table 1. Mean and median BMI and prevalence of overweight and obesity were higher in women than in men (p < 0.001). Mean FBG was similar in men and in women. Mean and median FSI were higher in women than in men (p < 0.001).

**Table 1:** Age-standardized means and medians of selected characteristics by sex

		Men		Women		All	
				
n		568		687		1255	
Age (years)							
25–34†	%	22.2		21.7		21.9	
35–44†	%	23.6		25.6		24.7	
45–54†	%	27.8		26.3		27.0	
55–64†	%	26.4		26.4		26.4	
Mean age†	years	45.3	(0.5)	45.1	(0.4)	45.2	(0.3)
Mean age	years	42.1	(0.5)	41.9	(0.4)	42.0	(0.3)
Mean body mass index	kg/m^2^	25.5	(0.2)	28.3	(0.2)	26.9	(0.2)
Median body mass index	kg/m^2^	25.2	(6.3)	27.8	(8.5)	26.4	(7.4)
Prevalence underweight	%	4.6	(0.9)	3.3	(0.7)	4.0	(0.6)
Prevalence excess weight	%	51.9	(2.1)	68.3	(1.9)	60.1	(1.4)
Prevalence obesity	%	15.0	(1.5)	35.1	(1.8)	25.0	(1.2)
Mean fasting blood glucose	mmol/l	6.0	(0.1)	5.7	(0.1)	5.9	(0.1)
Median fasting blood glucose	mmol/l	5.5	(0.8)	5.3	(0.7)	5.4	(0.8)
Mean fasting serum insulin	pmol/l	81.0	(3.1)	96.6	(2.8)	88.8	(2.1)
Median fasting serum insulin	pmol/l	63.0	(48.6)	76.2	(55.2)	69.6	(52.2)
Mean HOMA-IR		4.1	(0.2)	4.5	(0.2)	4.3	(0.1)
Median HOMA-IR		2.7	(2.4)	3.1	(2.7)	2.9	(2.5)

Standard errors and inter-quartile ranges are indicated between brackets for means and medians, respectively. Except for age, all estimates are standardized for age [19].

Underweight: BMI <18.5 km/m^2^;excess weight: BMI ≥ 25 km/m^2^; obesity: BMI ≥ 30 kg/m^2^.

HOMA-IR: homeostasis model assessment of insulin resistance.

† Crude estimates. Other estimates are standardized for age.

Table 2 shows the prevalence of different categories of impaired glucose regulation. The prevalence of DM was particularly high in the oldest age group (55–64) in both sexes. The prevalence of IFG was higher in men than in women (p < 0.001). Since the measurement of FBG alone may leave some DM persons undetected, an OGTT was performed in individuals with FBG between 5.6–6.9 mmol/l. Using OGTT results for the diagnosis of DM (i.e. 2hBG ≥ 11.1 mmol/l) in addition to other criteria for DM, the overall prevalence of DM increased by 2.1% (absolute difference) or 22% (relative difference). The prevalence of IGT did not differ significantly (p > 0.05) between genders. Of note, the prevalence of combined IFG/IGT is necessarily equal to the prevalence of IFG in our study since OGTT was performed in all subjects with IFG (i.e. FBG between 5.6 and 6.9 mmol/l). Based on all three criteria for DM, the prevalence of DM standardized for the actual population of Seychelles in 2004 (i.e. not adjusted to the WHO standard population) was 10.2% overall (95%CI: 8.6–11.9); men: 10.2% (7.7–12.7), women: 10.3 (8.0–12.6). The slightly lower prevalence using actual age distribution in Seychelles vs. the WHO age distribution reflects that the proportion of young vs. old people is slightly larger in the actual population of Seychelles than in the WHO standard age distribution.

**Table 2:** Prevalence of diabetes, impaired fasting glucose and impaired glucose tolerance (percent and 95% confidence interval) and proportion of diabetic persons aware of having diabetes

	Men				Women				25–64		
			
	25–34	35–44	45–54	55–64	25–34	35–44	45–54	55–64	Men	Women	All
n	126	134	158	150	149	176	181	181	568	687	1255
**Diabetes (DM)**											
DM (2 criteria)	0.8	9.7	12.7	22.0	2.0	4.6	11.6	26.5	9.6	9.1	9.4
	(0–2.3)	(4.7–15)	(7.5–18)	(15–29)	(0.2–2.3)	(1.5–7.6)	(6.9–16)	(20–33)	(7.4–12)	(7.2–11)	(7.9–11)
DM (3 criteria)	0.8	9.7	14.6	27.3	3.4	6.3	14.9	34.3	11.0	12.1	11.5
	(0–2.3)	(4.7–15)	(9.1–20)	(20–35)	(0.5–2.9)	(2.7–9.8)	(9.7–20)	(27–41)	(8.7–13)	(9.9–14)	(9.9–13)
**Impaired fasting glucose**											
IFG	16.0	35.1	42.4	32.9	5.4	13.6	26.5	37.0	30.4	18.0	24.2
	(9.6–22)	(27–43)	(35–50)	(25–40)	(1.7–3.6)	(8.6–19)	(20–33)	(30–44)	(27–34)	(15–21)	(22–26)
**Glucose tolerance**											
NGT (2hBG <7.8)	12.8	23.9	21.5	10.7	2.7	2.3	8.8	12.7	17.6	5.7	11.6
	(6.9–19)	(16–31)	(15–28)	(5.8–16)	(0.1–2.6)	(0.1–4.5)	(4.7–13)	(7.8–18)	(14–21)	(4.1–7.3)	(9.8–14)
IGT (2hBG 7.8–11.0)	3.2	10.5	18.4	17.5	2.0	9.7	14.4	17.1	11.2	9.6	10.4
	(0.1–6.3)	(5.3–16)	(12–24)	(11–24)	(0–2.3)	(5.3–14)	(9.2–20)	(12–23)	(8.7–14)	(7.5–12)	(8.8–12)
DM (2hBG ≥ 11.1)	0.0	0.8	2.5	4.7	0.7	1.7	3.3	7.2	1.6	2.7	2.1
		(0–2.2)	(0.1–5.0)	(1.3–8.1)	(0–1.3)	(0–3.6)	(0.7–5.9)	(3.4–11)	(0.7–2.5)	(1.6–3.8)	(1.4–2.9)
**Aware of DM (among DM)**											
	0.0	30.8	45.0	60.6	0.0	50.0	61.9	64.6	47.1	61.5	53.9
		(4.7–56)	(23–67)	(44–77)		(13–87)	(41–83)	(51–78)	(35–59)	(50–73)	(46–62)

FBG: fasting blood glucose; IFG: impaired fasting glucose; 2hBG: blood glucose 2 hours after oral tolerance test; NGT: normal glucose tolerance; IGT: impaired glucose tolerance; DM: diabetes mellitus.

DM (2 criteria): FBG ≥ 7.0 mmol/l or history of treatment for DM.

DM (3 criteria): FBG ≥ 7.0 mmol/l, history of treatment for DM, or 2hBG ≥ 11.1 mmol/l.

Oral glucose tolerance test was administered to all individuals with FBG of 5.6–6.9 mmol/l without previous history of diabetes.

Overall prevalence estimates are standardized for age [19].

Table 3 shows the odds ratios relating excess body weight to DM and the proportions of DM cases in the population that could be attributable to overweight and obesity, by sex and overall. Estimates are standardized to the WHO age distribution and regression models are also adjusted for age. The prevalence of overweight and obesity was higher in women than in men (p < 0.001). DM was strongly associated with excess body weight (e.g. OR in both men and women: 2.6 for BMI more vs. less than 25 kg/m^2^). The OR of DM associated with overweight and obesity appearing in the table were virtually unchanged if underweight (BMI <18.5 kg/m^2^, less than 6%) was also factored in the analysis (the reference BMI category being then 18.5–24.9 kg/m^2^). The proportion of all DM cases in the entire population that could be attributed to excess weight was 49% (95%: 35%–61%). By using lower cut off values to define the reference BMI category -as used in other studies [21]-, the proportions of all cases of DM in the population that are attributable to excess weight increased to 58% (95% CI: 56%–60%) for a BMI cut off set at ≥ 24 kg/m^2 ^and to 73% (71%–74%) for a BMI cut off set at ≥23 kg/m^2^.

**Table 3:** Relationship between categories of body mass index (BMI) and diabetes and proportion of diabetic persons in the entire population that is attributable to overweight and obesity (95% confidence intervals in brackets)

	Men			Women			All		
			
	Prevalence (%)	Odds ratio*	PAF (%)	Prevalence (%)	Odds ratio*	PAF (%)	Prevalence (%)	Odds ratio*	PAF (%)
Overweight									
(BMI: 25–29 kg/m^2^)	37 (33–41)	2.1 (1.9–2.2)	26 (16–36)	33 (30–37)	2.6 (2.3–3.0)	20 (12–26)	35 (32–38)	3.5 (3.2–3.8)	23 (14–31)
Obesity									
(BMI ≥ 30 kg/m^2^)	15 (12–18)	2.6 (2.4–2.9)	17 (11–23)	35 (32–29)	4.5 (4.0–5.1)	36 (25–45)	25 (23–28)	3.3 (2.0–5.5)	26 (15–36)
Overweight or obesity									
(BMI ≥ 25 kg/m^2^)	52 (48–56)	2.2 (2.1–2.4)	43 (29–55)	68 (64–72)	3.6 (3.2–4.1)	56 (41–67)	60 (57–63)	2.6 (2.4–2.8)	49 (35–61)

All models are adjusted for age.

Estimates are standardized for age [19].

BMI: body mass index.

PAF: population attributable fraction.

* reference category: BMI <25 kg/m^2^.

In a separate model, the odds for having DM were 2.4 (1.7–3.5) times higher in persons with family history of DM compared to those without it. This OR for family history of DM was virtually identical whether BMI was included in multivariate analysis or not and whether models were run in all participants, men or women. Adjusting for age, sex and BMI, the proportion of all DM cases in the population that could be attributable to family history of DM was 25% (14–35%).

**Figure 1:**
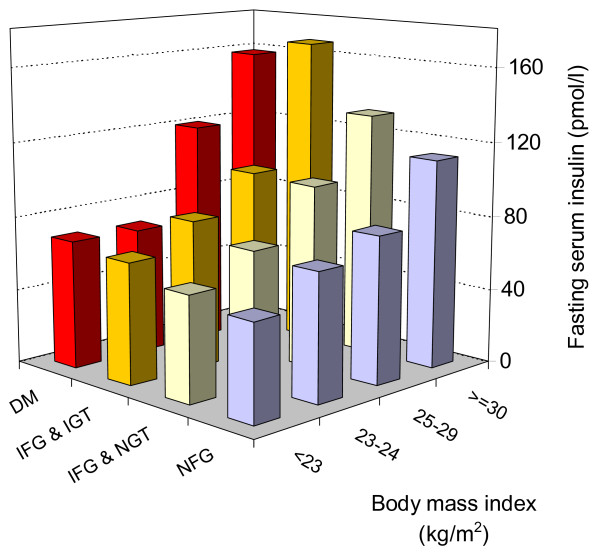
**Mean fasting serum insulin concentration by categories of body mass index (BMI) and categories of glucose metabolism impairment**. NFG: normal fasting glucose; IFG: impaired fasting glucose; NGT: normal glucose tolerance; IGT: impaired glucose tolerance; DM: diabetes mellitus (excluding patients on insulin treatment).

Figure 1 shows that mean FSI increased gradually across both categories of BMI and categories of impairment of glucose metabolism (i.e. NFG, IFG, IGT, DM). In separate analyses adjusted for age and sex (analyses not shown), the same relationships were found between FSI and both categories of impaired glucose regulation and BMI categories (p for trend < 0.001 for both). In these analyses, DM patients who were on insulin treatment (n = 10) were excluded. Analyses with HOMA-IR instead of FSI showed same patterns of association with categories of BMI and glucose metabolism impairment.

**Figure 2:**
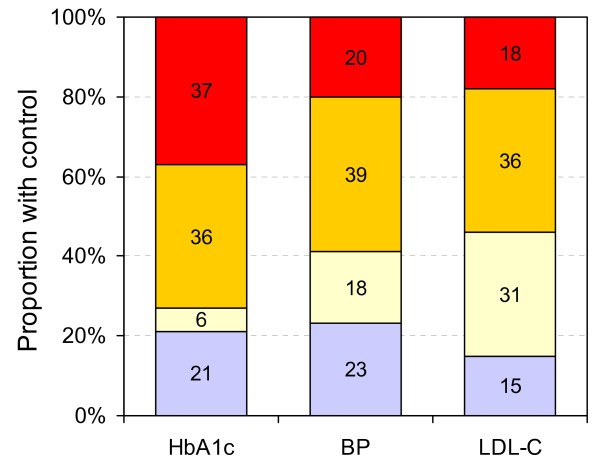
**Level of control of blood glucose (HbA1c: glycatedhemoglobin), blood pressure (BP) and low-density lipoprotein cholesterol (LDL-C) in all participants receiving hypoglycemic treatment (n = 80)**. Cut off values for control categories are <7.0, 7.0–7.9, 8–9.9, ≥ 10 for HbA1c; <130/80; 130-9/80-9; 140-59/90–99, ≥ 160/100 mmHg for BP; and <2.6, 2.6–3.4, 3.5–4.9, ≥ 5 mmol/l for LDL-cholesterol. Blue: within recommended treatment targets.

Figure 2 shows, among all persons reporting current anti-diabetic treatment (n = 80), the proportions who achieved different levels of blood glucose (based on HbA1c), BP and LDL-cholesterol. Less than a quarter of diabetic persons under treatment achieved recommended treatment targets for any of these three considered cardiometabolic conditions, i.e. HbA1c <7, BP <130/80 mmHg, and LDL-cholesterol <2.6 mmol/l. Less than 50% of treated patients achieved levels HbA1c <8, BP <140/90 mmHg and LDL-cholesterol <3.5 mmol/l. Almost 40% had HbA1c ≥ 10 and approximately 20% had BP ≥ 160/100 or LDL cholesterol ≥ 5 mmol/l. The prevalence of high blood pressure and hypercholesterolemia in this population were published earlier [22].

## Discussion

We found a high prevalence of DM in a rapidly developing country in the African region, a substantial proportion of persons unaware of DM in the population, limited cardiometabolic control among treated diabetic persons, and a strong association of DM with excess weight. These findings in Seychelles add to the few population-based data on DM available in the African region and may be representative of other countries experiencing rapid socio-economic development and concurrent lifestyle changes.

The prevalence of DM is higher in Seychelles than in predominantly rural African regions such as Nigeria [3], Cameroon [23] or Tanzania [24] but as high as in urban settings in South Africa [25] and Egypt [9]. The prevalence of DM in Seychelles is however lower than in the island of Mauritius [4] despite larger BMI in the population of Seychelles than in Mauritius. The difference between these two neighbor islands of similar economic development may partly relate to their different ethnic composition (predominantly African in Seychelles and predominantly Indian in Mauritius) since Indian descent is a known risk factor for DM [4,26]. Compared to non African countries, the prevalence of DM in the Seychelles is similar to estimates in urban Saudi Arabia [27], several regions of Europe [28], the United States [29] and urban India [6] but higher than in Mongolia [30], Bangladesh [31], the Philippines [32], Spain [33], Australia [34], Turkey [35] and rural Saudi Arabia [27].

A few factors in our study may tend to over or underestimate the true prevalence of DM in the population. Possible biases for underestimation are several. First we did not include individuals older than 64 years, an age group in which the prevalence of DM is likely to be particularly high [4,29,36,37]. Second, we did not perform an OGTT in individuals with FBG <5.6 mmol/l and we could have missed a few DM cases in persons with normal fasting glucose but pathologically high post meal levels. The number of such cases is however expected to be small [38]. Third, underestimation might also have occurred if non participation was related to diabetes-related diseases (e.g. leg wounds, renal failure, stroke, etc). On the other hand, overestimation may have occurred if some persons with elevated FBG were not fasting. This bias was minimized as persons who reported not to be fasting were invited to attend the survey on another day. Also, elevated fasting blood glucose was not repeated on a separate day. Overall, it is possible that factors that over or underestimate the prevalence of DM might balance each other and our prevalence estimates may be close to the true prevalence of DM in the adult population.

The high prevalence of IFG and/or IGT suggests that the DM epidemic has not yet plateaued in Seychelles. Indeed, IGT [39] and IFG [40] are strong predictors of DM [41] and these pre-diabetes conditions may occur in up to 60% of individuals several years before DM develops [42]. It has been reported that in the USA, where the prevalence of IFG and IGT is respectively 26% and 15%, approximately 25% of individuals with IFG/IGT will progress to diabetes, 50% will remain in their abnormal glycemic state, and 25% will revert to normal glucose tolerance (NGT) over a period of 3 to 5 years [43]. Hence, the substantially high proportion of pre-diabetes in Seychelles predicts a further increase in the prevalence of DM over the next few years, consistent with projections in other African countries in epidemiological transition [23,26,36].

While the proportion of all diabetes cases in the population who are aware of having DM ("aware") is approximately 50%, similar low proportions (about 50%) are also typically found in middle- or high-income countries such as Egypt [9], Saudi Arabia [27], Spain [33] and the U.S. [44]. Such low figures emphasize the difficulty to identify a disease (DM) that is most often silent for many years after onset.

We found that a large proportion of diabetic persons under anti-diabetic treatment had levels of blood sugar (as assessed by HbA1c), blood pressure, and LDL-cholesterol above the recommended therapeutic targets. Limited clinical control of DM is also found in high income western countries such as the USA [45]. In the USA, only 37% of DM patients had HbA1c levels <7.0%, only 35.8% achieved target blood pressure (<130/80 mmHg) and 51.8% of DM patients had hypercholesterolemia [45]. This further illustrates the great difficulty in achieving and sustaining good control of blood glucose, blood pressure and blood lipids in chronic diseases such as DM. In addition, limited cardiometabolic control is known to be even more difficult to achieve in DM patients who are overweight -a frequent occurrence- because of underlying insulin resistance [45,46].

DM was associated strongly with overweight, independent of age, sex and family history of DM. This relationship has been found consistently in other populations [27,32,33,35,47] and, for example, 90% of new DM cases among both African and white Americans had BMI ≥ 23 kg/m^2 ^[21]. In our study, half of all DM cases could be attributed to overweight or obesity. This proportion rose to 73% if normal weight was considered for BMI <23 kg/m^2 ^instead of BMI <25 kg/m^2^. In a Taiwanese cohort as much as 71% of DM cases were attributable to a BMI ≥ 25 kg/m^2^[48].

It is important to attempt to determine if the prevalence rates of DM and overweight (i.e. "diabesity") has increased over time in order to anticipate epidemiological trends and inform health care policy. The prevalence of DM was assessed for the first time in 1989 in the population of Seychelles [49] and it was found that 3.4% of men and 4.6% of women had DM. Using the same criteria in 1989 and 2004 (i.e. known diabetes and/or elevated fasting blood glucose) and the same age standardization (new WHO age distribution [20]) the prevalence of DM significantly increased between the two years in men (from 6.2% to 9.6%) and in women (from 6.1% to 9.1%). Since the survey methods were not identical in 1989 and 2004 (e.g. glucometers), there is a degree of uncertainty in these trend estimates. However, a true increase in the prevalence of DM over time is consistent with the large increase in obesity in men (from 4.3% to 15.0%) and women (from 27.9% to 35.1%). Increasing prevalence of overweight/obesity in the population in the interval is likely related to increasingly sedentary behaviors and larger caloric intake. Currently, more than 75% of workers are employed in services (vs. only 20% in industry and 5% in agriculture) [11,12]. The number of both private cars and passengers transported by public buses has doubled in the past 10 years (figures from the Licensing Authority and the Seychelles Public Transport Company, respectively). On the other hand, food balance sheets indicate that calorie availability per capita has increased substantially in Seychelles, e.g. from 1800 kcal in 1965, 2300 kcal in the late 1980s, and above 2400 kcal in the early 2000s [50]. The proportion of carbohydrates has decreased over time (74% of total calories in 1965 and 55% in 2000) while the proportion of fats has increased (16% in 1965 and 32% in 2000) [50]. The production of carbonated soft drinks by the main local manufacturer has tripled in the past 25 years (figures from Seychelles Breweries Ltd).

## Conclusion

The prevalence of DM in Seychelles has reached or exceeded levels typically found in several middle- or high-income countries. The strong association between DM and excess body weight emphasizes the importance of weight control interventions at a population level as a cornerstone strategy to curb the "diabesity" epidemic [51]. From a clinical perspective, the substantial proportion of persons unaware of having DM calls for improved early detection of diabetic persons. The high proportion of treated diabetic persons with insufficient cardiometabolic control stresses the need for intensifying clinical care to diabetic patients in order to minimize complications [52].

## Abbreviations

DM: diabetes mellitus; FBG: fasting blood glucose; IFG: impaired fasting glucose; IGT: impaired glucose tolerance; HOMA: homeostasis model assessment of insulin resistance; 2-hour OGTT: oral glucose tolerance test; 2hBG: 2-hour postload blood glucose; BP: blood pressure; LDL-C: low-density lipoprotein cholesterol; HbA1c: glycated haemoglobin; PAF: population attributable fraction.

## Competing interests

The author(s) declare that they have no competing interests.

## Authors' contributions

DF lead the analysis of the data and the write up of the manuscript. JW coordinated several aspects of the survey and reviewed the manuscript. LT performed insulin assays and reviewed the manuscript. ER assisted in the interpretation of data and reviewed the manuscript. PB lead the organization of the survey, assisted with the analysis and interpretation of the data and with the write up of the manuscript. All authors read and approved the final manuscript.

## Pre-publication history

The pre-publication history for this paper can be accessed here:


